# Supplementary material for: DMRforPairs: identifying Differentially Methylated Regions between unique samples using array based methylation profiles
Source: BMC Bioinformatics. 2014 May 15;15:141. doi: 10.1186/1471-2105-15-141 (PMC4046028; doi:10.1186/1471-2105-15-141)
Supplement: Additional file 2 — DMRforPairs output for the comparison of A431-MCF7 and NA17018-NA17105. Please start from the HTML files in each folder. Available via the BMC Bioinformatics website. [file 1471-2105-15-141-S2.zip › 1394847754114233_MOESM2_ESM/A431_MCF7/figures/19593.pdf]

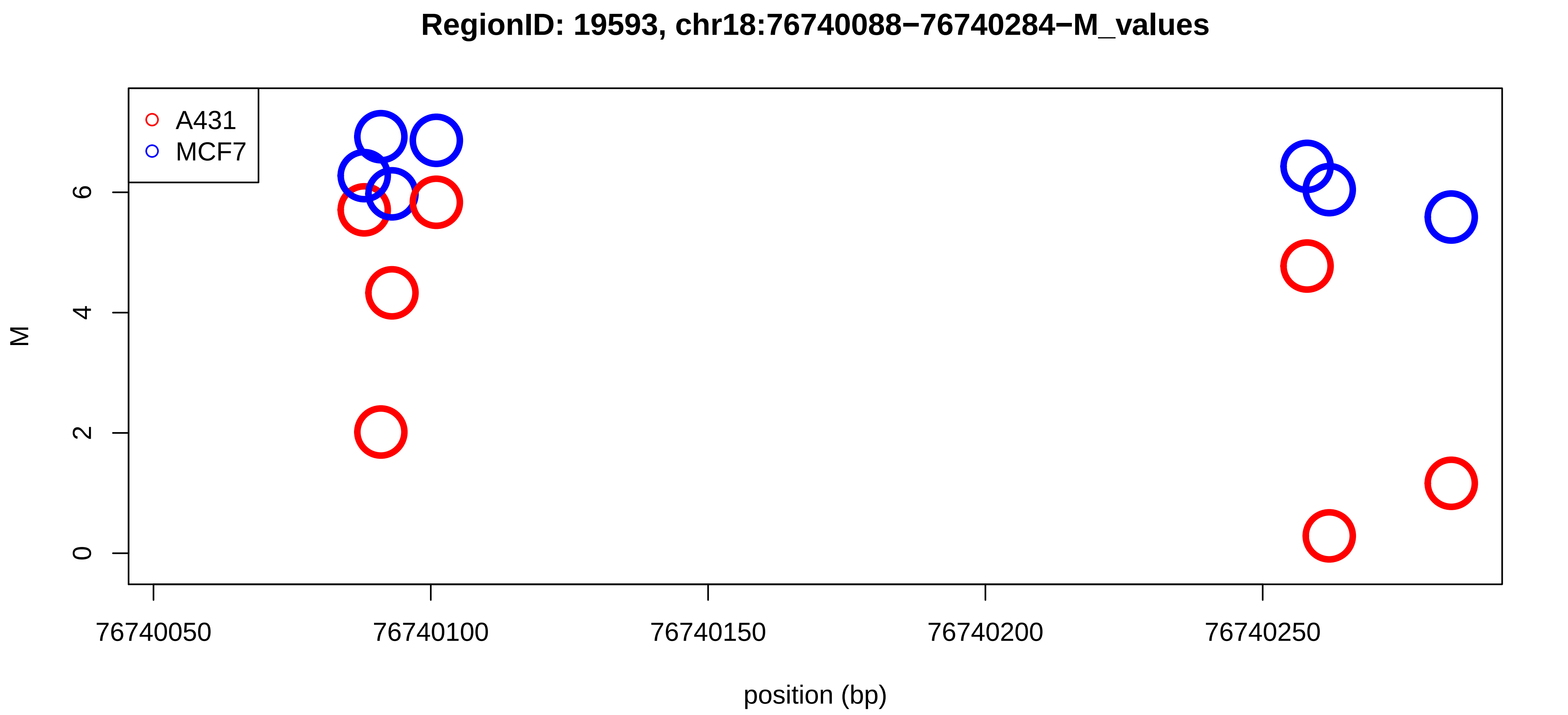
RegionID: 19593, chr18:76740088−76740284−M_values
A431
MCF7
M
0
2
4
6
76740050
76740100
76740150
76740200
76740250
position (bp)

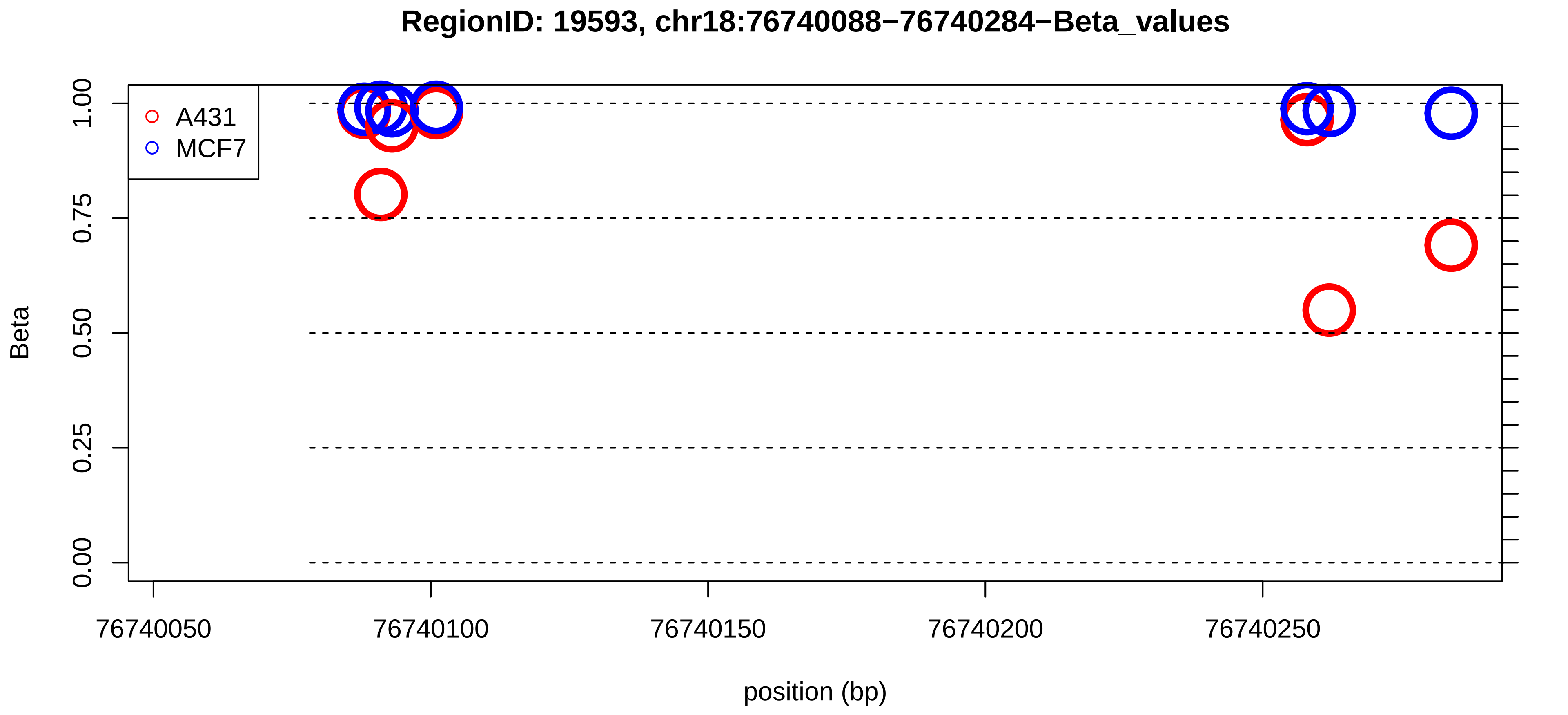
RegionID: 19593, chr18:76740088−76740284−Beta_values
A431
MCF7
Beta
0.00
0.25
0.50
0.75
1.00
76740050
76740100
76740150
76740200
76740250
position (bp)

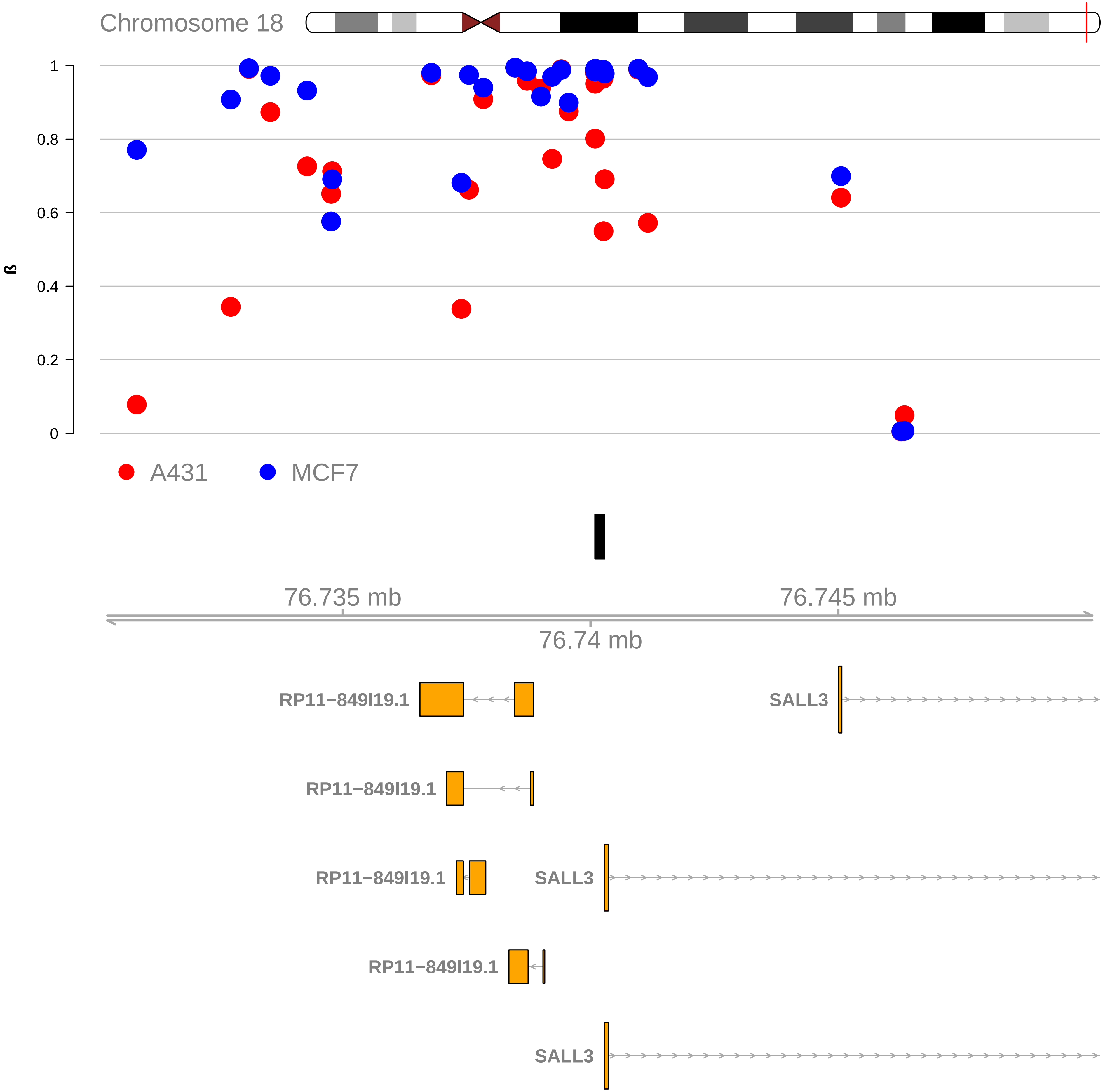
Chromosome 18
1
0.8
0.6
ß
0.4
0.2
0
A431
MCF7
76.735 mb
76.745 mb
76.74 mb
RP11−849I19.1
SALL3
RP11−849I19.1
RP11−849I19.1
SALL3
RP11−849I19.1
SALL3
